# A Thick-film Sensor as a Novel Device for Determination of Polyphenols and Their Antioxidant Capacity in White Wine

**DOI:** 10.3390/s100301670

**Published:** 2010-03-02

**Authors:** Kanokorn Photinon, Yongyuth Chalermchart, Chartchai Khanongnuch, Shih-Han Wang, Chung-Chiun Liu

**Affiliations:** 1 Faculty of Agro-Industry, Chiang Mai University, Muang, Chiang Mai 50100, Thailand; E-Mails: ychalermchart@gmail.com (Y.C.); aiickhnn@chiangmai.ac.th (C.K.); 2 Chemical Engineering Department, I-Shou University, 1, Sec. 1, Syuecheng Rd., Dashu Township, Kaohsiung County 840, Taiwan; E-Mail: shwang@mail.isu.edu.tw; 3 Chemical Engineering Department, Case Western Reserve University/10900 Euclid Ave., Cleveland OH 44106, USA; E-Mail: cxl9@case.edu

**Keywords:** thick-film, polyphenols, antioxidant capacity, caffeic acid

## Abstract

A thick-film electrochemical sensor with an iridium-carbon working electrode was used for determining polyphenols and their antioxidant capacity in white wine. Caffeic acid was used as a model species because it has the ability to produce the highest oxidation current. The correlation coefficient of 0.9975 was obtained between sensor response and caffeic acid content. The total phenolic content (TPC) and scavenging activity on 1,1-diphenyl-2-pycrylhydrazyl (DPPH·) radical were also found to be strongly correlated with the concentration of caffeic acid, with a correlation coefficient of 0.9823 and 0.9958, respectively. The sensor prototype was proven to be a simple, efficient and cost effective device to evaluate the antioxidant capacity of substances.

## 1. Introduction

Oxidative stress is a pathological condition associated with cell damage from excessive production of peroxides and free radicals. Vast epidemiological studies have shown a correlation between oxidative stress and non-infectious diseases such as cancer, cardiovascular disease, aging and neurodegenerative diseases [1]. Polyphenolic compounds or polyphenols possess both hydroxyl groups and the potential for delocalization of pi-electrons within the individual ring [2], which allow them to act as antioxidants, effectively rendering the oxidative properties of free radicals. There is evidence that indicates a diet rich in polyphenols may provide a positive effect on reducing oxidative stress due to their antioxidant properties [3,4]. Wine is widely recognized as one of the most important sources of polyphenols, and thus a moderate consumption of wine is considered to be a health benefit. Consequently, the assessment of the antioxidant capacity of wine and other polyphenol-rich diets has been of great interest.

The antioxidant capacity can be quantified using various methods differing in terms of their assay principle and experimental conditions. The methods basically determine the ability to reduce free radicals of polyphenols. Due to the complexity of the free radical-scavenging mechanisms, the antioxidant capacity cannot entirely be assessed using a single technique. The two highly correlated and widely used assays for wines are scavenging activity on 1,1-diphenyl-2-pycrylhydrazyl radical (DPPH·) and total phenolic content (TPC) [5,6]. The former method employs the stable DPPH· whose spectrum is in the UV-Vis range. Upon adding antioxidants, the absorbance of DPPH· is diminished and thus reveals the antioxidant capacity of the polyphenols [7]. The latter technique, misleading by the name “total phenolic content,” actually measures the reducing capacity of the sample via colorimetry using Folin-Ciocalteau reagent, which contains oxidants that change color upon being reduced [8,9]. Nevertheless, the colorimetric technique sometimes involves sample preparation and procedures that are either time or labor consuming or extensive chemical usage. Owing to rapid response, cost-effectiveness, simplicity of operation, and minimal solvent requirements, thick-film electrochemical sensors evidently provide an alternative approach to overcoming such limitations [10–12]. Although there is a growing interest on employing electrochemical sensors for polyphenol determination in wine [13–18], the reports on its correlation with the conventional assays is still very limited.

The objective of the present work was to investigate the possibility to use a thick-film screen-printed electrochemical sensor as a novel device for determination of polyphenols and their antioxidant capacity in white wine. The conventional techniques for measuring antioxidant capacity *i.e.,* scavenging activity on DPPH·and total phenolic content, were also performed to study the feasibility of using the electrochemical sensor as an alternative approach. The correlations among those techniques were then assessed.

## 2. Results and Discussion

### 2.1. Determination of Caffeic Acid in White Wine Using Electrochemical Sensor

**Figure 1.**
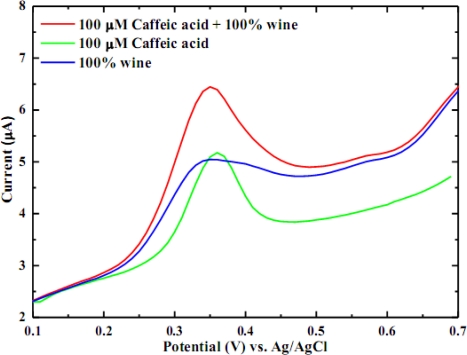
Voltammograms of caffeic acid, wine and caffeic acid-added wine showing that the most electroactive species in white wine is caffeic acid.

The phenolic composition of wines is conditioned by grape variety, geographical location and winemaking technology. However, the major polyphenols in wine are gallic acid, catechin, epicatechin, p-cumaric acid and caffeic acid [6,19,20]. It was found that caffeic acid is one of the most electrochemically active among the major pholyphenols [14,16,17] and hence was chosen to be the model species for polyphenolic compound determination in white wine. The cyclic voltammogram of standard caffeic acid solution and white wine were performed in order to identify the oxidation potential for caffeic acid in white wine. It was anticipated that caffeic acid would produce the highest oxidizing current and hence be the most pronounced peak in white wine. It is clearly shown in Figure 1 that the most pronounced peak in white wine is due to caffeic acid. The voltammogram of wine alone has an oxidation peak at +0.35 V (*vs.* Ag/AgCl) and this peak was promoted once caffeic acid was added, emphasizing the presence and domination of caffeic acid in white wine. In order to further confirm the previous finding, the caffeic acid voltammogram was compared with a voltammogram of wine-added caffeic acid. The oxidation current for caffeic acid increased upon adding wine. Therefore, it would be reasonable to use caffeic acid as a model species for polyphenols in white wine. The calibration curve for caffeic acid (not shown) possesses a sensitivity of 0.10643 μA/(mg/L) with a linear correlation of 0.9975 and linear range of 0.0–25.0 mg/L.

**Figure 2.**
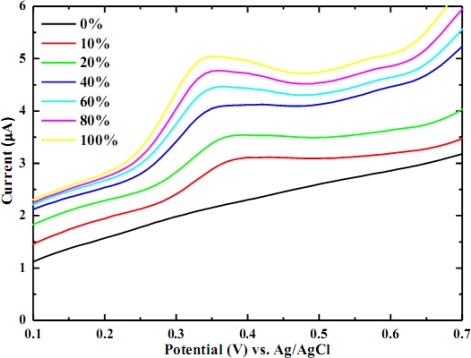
Voltammograms of wine sample at different dilution ratios.

It is customary to dilute the real sample prior to electrochemical analysis because the real sample is generally a complex matrix of various constituents that can possibly undergo oxidation, and contribute background current, hence lessening the signal-to-noise ratio. Precaution needs to be taken when selecting the dilution ratio to avoid the non-linear response. For the wine sample, a dilution ratio of 10 times is required for the glassy carbon electrode [16] and 250–2000 times for the platinum electrode [15]. The precious metals usually possess a very high activity. Most species are readily oxidized at relatively low potential on platinum, whereas, the oxidation potential for the glassy carbon electrode is higher, thus enabling the undesirable co-oxidation of interference. Metalized carbon (metal particles dispersed carbon) has shown the ability to oxidize electroactive species at low potential that minimizes background current and hence favors the signal-to-noise ratio [12,21,22]. The working electrode in this work is iridium-carbon (Ir-C), which has an oxidizing potential of +0.35 V (*vs.* Ag/AgCl), while glassy carbon oxidizes caffeic acid at +0.45 V [16]. Consequently, it is anticipated that the response is linear over the entire concentration range and the dilution is not necessary for the white wine sample as it is shown in Figure 2. The calibration curve for various proportions of wine (not shown) possesses a sensitivity of 0.0116 μA/(% wine) with a linear correlation of 0.9928.

### 2.2. Total Phenolic Content (TPC) and Scavenging Activity on DPPH

**Figure 3.**
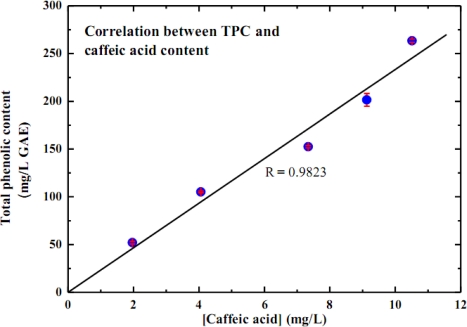
Correlation between total phenolic content and caffeic acid content.

**Figure 4.**
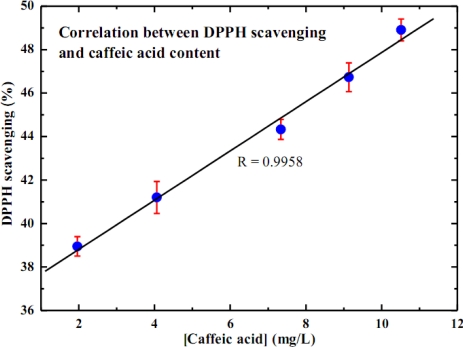
Correlation between %DPPH·scavenging and caffeic acid content.

The antioxidant capacity of substances is conventionally characterized using the scavenging activity on DPPH· and total phenolic content assays. Thus, the correlations between caffeic acid content and those parameters are essential in order to justify the electrochemical sensor as an alternative approach. Figure 3 illustrates a linear correlation of 0.9823 between TPC and caffeic acid content. A linear correlation of 0.9958 against % DPPH· scavenging is reported in Figure 4. Since it is the major polyphenol in white wine, caffeic acid therefore presumably contributes most of the antioxidant capacity of white wine. Consequently, caffeic acid content could legitimately reflect the antioxidant capacity of white wine.

## 3. Experimental Section

### 3.1. Chemicals and Instruments

Caffeic acid, Folin-Ciocalteu reagent, and 2,2-diphenyl-1-picryldyfrazyl radical (DPPH·) were purchased from Sigma-Aldrich. The potentiostat is the CHI405 Electrochemical Work Station (CHI instrument, Austin, Texas, USA). White wine under the trademark “Michel Torino” year 2007 from Argentina was purchased from a local store.

### 3.2. Determination of Caffeic Acid in White Wine Using Electrochemical Sensor

**Figure 5.**
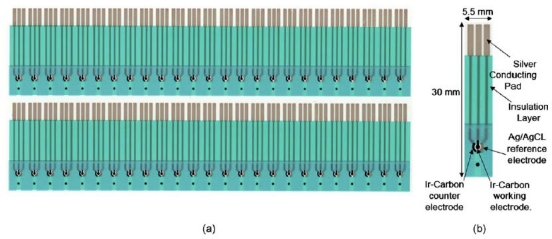
Sensor layouts in two rows (a) and detailed configuration (b) [12].

The disposable mini sensors were fabricated in bulk using screen-printing technology on a polyester substrate (Conductive Technology, York, Pennsylvania, USA). Figure 5 shows two rows of the mini sensors (a) and a detailed configuration (b). Each sensor comprised of three electrodes: iridium-containing carbon (Ir-C)working and counter electrodes, and an Ag/AgCl reference electrode. The diameter of Ir-C working electrode was approximately 1 mm.

White wine usually contains approximately 13.5% vol/vol of alcohol and has pH in the range of 3.4–3.6, the medium for caffeic acid was thus prepared in the similar manner in which hydrochloric acid 0.025 mM was used to adjust the pH. The calibration curve of caffeic acid was prepared in the range of 0.0–25.0 mg/L and was generated using cyclic voltammetry with the scan rate of 50 mV/s. Caffeic acid content in 6 different dilution ratios of white wine was also measured in order to determine the optimum dilution ratio.

### 3.3. Determination of Total Phenolic Content (TPC) and Scavenging Activity on DPPH·

The total phenolic content in white wine was determined according to the Folin-Ciocalteu colorimetric method [8,9]. The wine sample was mixed thoroughly with Folin-Ciocalteu reagent and its absorbance was measured at 765 nm, and the total phenolic compound was expressed as a gallic acid equivalent (mg/L GAE). The free radical-scavenging activity of wine was measured via spectrometry as well [7]. The wine sample was added to the methanolic DPPH· and the absorbance at 515 nm was determined. The decrease of absorbance at 515 nm upon adding the wine sample was then used to calculate %DPPH· scavenging.

## 4. Conclusions

Our thick-film screen-printed micro sensors have proven to be a promising approach for the characterization of white wine for its antioxidant capacity with rapid response, cost-effectiveness, simplicity of operation, and minimal solvent requirement. The sensor response was linearly correlated to caffeic acid content. Being one the most electroactive and the major polyphenol in white wine, caffeic acid was employed as a model phenolic compound. The investigation revealed that caffeic acid content possessed a high linear correlation with the most recognized assays to assess antioxidant capacity of substance *i.e.,* total phenolic content and DPPH· scavenging ability with the linear correlation of 0.9823 and 0.9958, respectively. Unlike DPPH· scavenging and total phenolic content assays that are usually measured spectrometrically and sometimes with involved sample preparations, electrochemistry requires minimal sample preparation and a shortened operation time. Furthermore, the iridium-containing carbon (Ir-C) working electrode possesses the ability to lower the oxidation potential, hence enhancing the signal-to-noise ratio and allowing the measurement of a wine sample without any dilution.

The advantages of this novel device will not be limited to characterizing wine; the investigation of utilizing the electrochemical sensors to other food substances will be carried out in the near future and strong correlations between sensor response and the conventional assays are anticipated.
